# New Work on Physiology

**Published:** 1838-10

**Authors:** W. B. Carpenter


### NEW WORK ON PHYSIOLOGY. BY W. B. CARPENTER.

We are happy to perceive, by our advertising columns, that Mr. Carpenter, of whose high talents and acquirements we have more than once had occasion to speak, has in the press a new work on Physiology, on an extended scale, and on an entirely new plan. We shall be greatly surprised if this work is not found admirably calculated to fill an important blank in physiological science and literature, which has been long deplored, as well by the medical reader as the man of science. The work, we understand, is designed to embrace all the general laws which modern physiological research has attained; and to illustrate their application to every class of organized beings. In the Introduction will be comprised an account of Organized Structures in general, a description and comparison of the elementary tissues of Plants and Animals, and an account of the structure and characters of the principal natural groups of living beings. Under the head of General Physiology will be embraced the laws of vital action, and of the development of organized structures so far as these are yet ascertained. The department of Special Physiology will be devoted to the consideration of each function in detail; the evolution of its organ, from its simplest and most general type to its most complex and specialized form, will be traced through the ascending scale, first of the vegetable and then of the animal kingdom, and in the fœtal development of the higher classes; and a general view of the function will then be given, and the varieties in its performance in the different classes will be reconciled with its fundamental uniformity in all. The work will be illustrated with six copper-plates, as well as with several woodcuts; and we understand that it has been the aim of the author to render it intelligible (by explanation and illustration of technical language) to the general reader, as well as to the professional student.

